# Correction to: Not so hidden anymore: Advances and challenges in understanding root growth under water deficits

**DOI:** 10.1093/plcell/koae150

**Published:** 2024-06-05

This is a correction to: Priya Voothuluru, Yajun Wu, Robert E Sharp, Not so hidden anymore: Advances and challenges in understanding root growth under water deficits, *The Plant Cell*, Volume 36, Issue 5, May 2024, Pages 1377–1409, https://doi.org/10.1093/plcell/koae055

The following changes have been made to the originally published manuscript.

Figure 4 has been updated to indicate decreased abundance under water stress in panel E. Figure 4 now reads:

**Figure:**
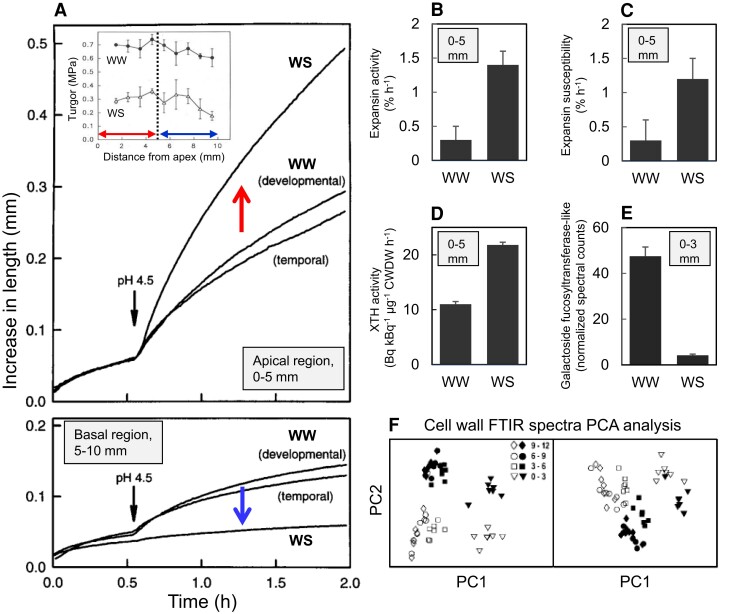


instead of:

**Figure:**
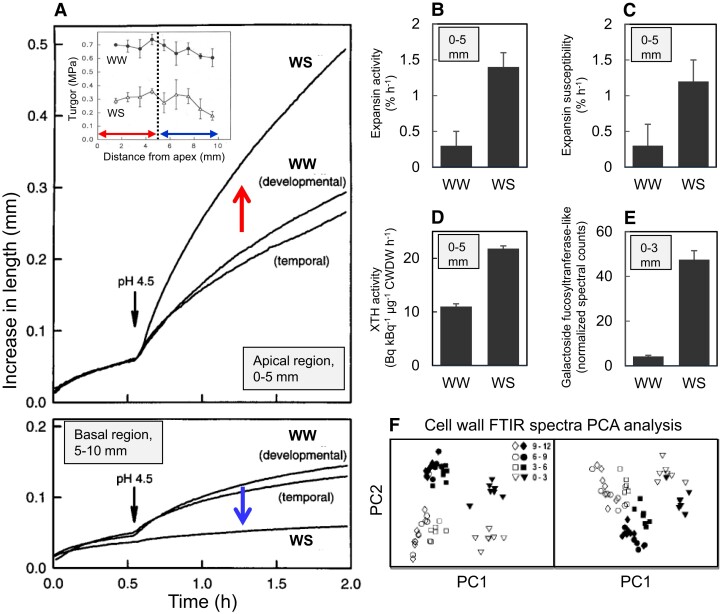


The legend of Figure 4 is also emended. The last part of the fifth sentence now reads: “[…], and **(D)** XTH activity per unit of cell wall dry weight (CWDW), as well as **(E)** decreased abundance of galactoside 2-α-1-fucosyltransferase-like protein.” instead of: “[…], **(D)** XTH activity per unit of cell wall dry weight (CWDW), and **(E)** abundance of galactoside 2-α-1-fucosyltranferase-like protein.”

